# Utility of Platelet Indices as a Predictive Marker in Sepsis: An Observational Study From North East India

**DOI:** 10.7759/cureus.38095

**Published:** 2023-04-25

**Authors:** Dibya J Sharma, Suman Ganguly, Rakesh M, Akash Batta, Abhishek Paul Majumder

**Affiliations:** 1 Internal Medicine, Gastroenterology, Silchar Medical College and Hospital, Silchar, IND; 2 Internal Medicine, Silchar Medical College and Hospital, Silchar, IND; 3 Cardiology, Dayanand Medical College and Hospital, Ludhiana, IND; 4 Community Medicine, Lokmanya Tilak Municipal Medical College and General Hospital, Mumbai, IND; 5 General Medicine, Agartala Government Medical College and GB Pant Hospital, Agartala, IND

**Keywords:** infection., sofa score, mortality, mean platelet volume, thrombocytopenia, sepsis, prognosis, affordable, observational, platelet indices

## Abstract

Background

Unraveling sepsis remains the holy grail of clinical medicine and the commonest cause of in-hospital mortality worldwide. Various newer biomarkers have emerged in recent years that aid in the diagnosis and prognostication of sepsis. However, the widespread use of these is limited by availability, cost, and long turnaround times. Considering the crucial role of hematological parameters in infectious conditions, the present study aimed to evaluate the association of various platelet indices with the severity and outcomes in patients diagnosed with sepsis.

Methods

This was a single-center, prospective, observational study comprising 100 consecutive patients who fulfilled the selection criteria in the emergency department of a tertiary care hospital from June 2021 to May 2022. All patients underwent history taking, physical examination, and necessary laboratory investigations, including complete blood counts, biochemistry panel, and radiographic and microbiological tests. A detailed assessment of various platelet indices (platelet count, mean platelet volume, and platelet distribution width) was performed, and its association with outcomes was derived. The Sequential Organ Failure Assessment (SOFA) score was recorded for all patients.

Results

The majority of the study population was male (52%) with a mean age of 48.05±19.27 years. Respiratory infection (38%) was the most common origin of sepsis followed by genitourinary infections in 27%. The mean platelet count on admission was 1.83±1.21 lakhs/mm^3^. The incidence of thrombocytopenia (<1.5 lakhs/ mm^3^) in our study sample was 35%. The overall in-hospital mortality of the study group was 30%. Thrombocytopenia was significantly associated with a higher SOFA score (7.4±3 vs. 3.7±1.9, P<0.05), longer hospital stays (10.8±4.6 vs. 7.8±3.9; p<0.05), and mortality (17 vs. 13; p<0.05). The change in platelet count, platelet distribution width, and mean platelet volume from Day 1 to Day 3 also correlated with outcomes. There was a decrease in platelet count among the non-survivors compared to an increase in platelet count among survivors from Day 1 to Day 3 (p<0.05). Similarly, the change in platelet distribution width showed a decreasing trend among the survivors compared to an increasing trend among the non-survivors (p<0.05). The mean platelet volume of non-survivors increased from Day 1 to Day 3 compared to a downward trend among the survivors (p<0.05).

Conclusion

Septic patients with thrombocytopenia on admission had a higher SOFA score and were associated with worse outcomes. Additionally, platelet indices, such as platelet distribution width and mean platelet volume, serve as important prognostic markers among sepsis patients. Change in these parameters from Day 1 to Day 3 also correlated with outcomes. These indices are simple and affordable, allowing for their serial assessment to aid in the prognosis of sepsis.

## Introduction

Sepsis is the second leading cause of death globally and a significant contributor to morbidity and mortality in low-income countries. Sepsis occurs when a person's body has an overwhelming response to an infection. Three pathways in the body are affected during sepsis: the inflammatory pathway, the circulation pathway, and the coagulation pathway. In sepsis, these pathways are dysfunctional, which leads to end-organ damage. If left untreated, sepsis leads to death. Early identification and intervention are imperative in sepsis. This is especially true in low-income countries, where resources are often limited [1-3].

Thrombocytes (platelets) are an important cellular component of the circulatory system and coagulation pathway. Thrombocytopenia (platelet count <1.5 lakhs/mm^3^), which accounts for 40% of coagulation disorders, is a frequent and multifactorial complication of sepsis [4]. The Sequential Organ Failure Assessment (SOFA) score is a measure of organ severity, and platelet count is a component of it [5-6]. The pathology behind thrombocytopenia is ‘platelet consumption’ by thrombin-mediated platelet activation, which can turn into disseminated intravascular coagulation (DIC) in severe cases [5-8]. Some of the commonly used platelet markers include platelet count, mean platelet volume (MPV), and platelet distribution width (PDW) [9].

Considering the crucial role of platelets in infectious conditions, the present study aimed to evaluate platelet indices, including platelet count, morphology, and turnover rate, along with the severity of sepsis and its associated morbidity and mortality.

## Materials and methods

Study design and setting

This was a hospital-based prospective observational study conducted at the inpatient department (IPD) of a tertiary care hospital, Silchar Medical College and Hospital (SMCH), in Assam in North East India from June 2021 to May 2022.

Study participants, inclusion, and exclusion criteria

All patients admitted during the study period in the inpatient department (IPD) of the medicine department of SMCH with clinical signs and symptoms suggestive of sepsis viz. tachypnoea, tachycardia, altered sensorium, along with infective foci, were evaluated for enrolment/eligibility in the study. Patients were included based on the following criteria: age 18 years or above and SOFA score ≥ 2. Patients were excluded based on the following criteria: 1) age less than 18 years, 2) pregnancy, 3) suspicion of drug-induced thrombocytopenia or recent intake of any drug that might cause thrombocytopenia, 4) patients who underwent radiotherapy, chemotherapy, or platelet transfusion in last 30 days; none of the patients in our study received platelet transfusion; 5) diagnosed or suspected cases of hematological disorder e.g., leukemia, immune thrombocytopenic purpura, etc., 6) chronic liver disease, 7) chronic renal failure, 8) alcohol abuse, and 9) admission time of less than 24 hours.

Sample size and sampling

In this study, all consecutive patients who fulfilled the inclusion criteria were selected. A total of 240 patients were evaluated in the study, out of which 94 patients were excluded, as they did not fulfill the inclusion criteria or had any of the exclusion criteria. Another 46 patients had not given consent for inclusion in the study. A total of 100 patients from the IPD after considering inclusion and exclusion criteria were included in the study for analysis. The evaluation of patients with sepsis is shown in Table 1. Prior to recruitment, consent was obtained from all the patients or from family members accompanying the patient if the patient was unable to give consent. After initial treatment and stabilization, a detailed history-taking and clinical examination was carried out followed by laboratory investigations, e.g., complete blood count (CBC), random blood sugar (RBS), kidney function test (KFT), liver function test (LFT), and routine urine examination. Arterial blood gas (ABG) and blood culture were done in all cases, and we repeated the platelet count once every 48 hours till discharge or death. Necessary radiological examinations like chest X-ray, computed tomography (CT), body fluid cultures, and electrocardiography (ECG) were done in selected cases to localize the foci of infection. Calculation of the SOFA score was done by measuring platelet count, serum bilirubin, creatinine, urine output, mean arterial pressure (MAP), Glasgow coma scale (GCS), and arterial oxygen pressure (PaO2)/fraction of inspired oxygen (FiO2). The patient's sociodemographic profile, clinical profile, and laboratory and radiological results were noted. Platelet indices included were platelet count (number of platelets/mm^3^), mean platelet volume in femtolitres (fL), and platelet distribution width in femtolitres (fL).

**Table 1:** Chart showing the evaluation of patients with sepsis

Total Patients Evaluated (240)		
Patients Included in the study (100)	Patients excluded (94)	Didn’t give consent (46)

Statistical analysis

Data were entered into a Microsoft Excel spreadsheet (Microsoft Corporation, Redmond, WA) and analysis was done using IBM SPSS (version 26, trial version; IBM Corp. Armonk, NY). Data were tested for normal distribution. Quantitative data were expressed as mean (±standard deviation) and categorical variables were summarized as frequencies with percentages. The student’s t-test/analysis of variance (ANOVA) and the chi-square test were applied as tests of significance. A p-value of <0.05 was considered significant in this study.

Ethical consideration

The institutional ethical committee of Silchar Medical College approved the study, and the ethical clearance number of the study was SMC/13/2020. Before data collection, informed written consent was taken in the patient’s own language.

## Results

Among the 100 patients included in the study, the majority were male (52%) with a mean age of 48.05±19.27 years. Respiratory infection (38/100) was the most common origin of sepsis followed by genito-urinary (27/100) and intra-abdominal (21/100) infections. Two major causes of sepsis among our study participants were pneumonia (23/100) and urinary tract infection (UTI) (18/100). It was observed that 35% of the cases had thrombocytopenia. The mean platelet count on admission was 1.83±1.21 lakhs/mm^3^. The baseline characteristics of the patients are shown in Table 2.

**Table 2:** Baseline characteristics of the patients

Age group (in years)	Frequency	Percentage
<20	7	7%
21-40	34	34%
41-60	29	19%
>60	30	30%
Gender	Frequency	Percentage
Male	52	52%
Female	48	48%
Etiology of sepsis	Frequency	Percentage
Respiratory infection	38	38%
Genito-urinary infection	27	27%
Abdominal infection	21	21%
Central nervous system infection	9	9%
Soft tissue infection	5	5%
Platelet count	Frequency	Percentage
Normal	65	65%
Thrombocytopenia	35	35%
Outcome	Frequency	Percentage
Died	30	30
Survived	70	70

Hospital stay was longer among patients who had thrombocytopenia (10.8±4.6 days) compared to those who had a normal platelet count (7.8±3.9 days), and this difference was found to be statistically significant (p-value <0.001). We also found a significant association between platelet count and patient outcomes as depicted in Table 3.

**Table 3:** Association between thrombocytopenia and outcomes SOFA: Sequential Organ Failure Assessment; PaO2: arterial oxygen pressure; FiO2: fraction of inspired oxygen

Parameter	Platelet Normal	Thrombocytopenia	p-value
SOFA Score	3.7±1.9	7.4±3.1	0.001
PaO_2_/ FiO_2_	370.54±90.2	340.7±98.1	0.129
Length of Hospital Stay ( Days)	7.8±3.9	10.8±4.6	0.001
Mortality	13	17	0.003
Alive	52	18	0.003

The mean SOFA score among patients with thrombocytopenia (7.4 ±3.1) was double what was seen in patients with normal count (3.7±1.9), which was statistically significant (p-value<0.001). This is obvious with the multisystem and coagulation disorders during septicemia. PaO2/FiO2 showed a slightly higher mean value in patients with normal platelet count (370.54±90.2 mmHg) compared to patients with thrombocytopenia (340.7±98.1 mmHg) without any association established (p-value=0.129). Similarly, mean serum bilirubin was comparatively higher among patients with thrombocytopenia without any significance (p-value=0.402). The SOFA score was statistically significant to the patient’s outcome (p-value <0.001). There was a decreasing trend of mean platelet count among the non-survivors and an increasing trend was seen among the survivors from Day 1 to Day 3. This shows how important it is to collect and send blood specimens daily for patients in the IPD with sepsis or any other infection. The mean platelet distribution width showed a decreasing trend among survivors and an increasing trend among the deceased from Day 1 to Day 3 with a p-value of <0.001. The mean platelet volume of the patients who did not survive showed an upward trend after admission, whereas a downward trend was seen among the alive ones (p-value<0.001) as shown in Table 4.

**Table 4:** Relation between platelet indices, SOFA score, and outcomes SOFA: Sequential Organ Failure Assessment

Platelet indices	Day	Survivors	Non-survivors	P value
Platelet count (Lakhs/mm^3^.) (Mean ± SD)	D1	205942.86 (±125789.41)	129883.33 (±90942.92)	0.004
	D2	203328.57 (±123446.02)	92233.33 (±65400.46)	<0.001
	D3	220811.59 (±123928.97)	106250.00(±48432.18)	<0.001
Platelet distribution width (fl) (Mean ± SD)	D1	9.75 (±1.99)	9.92(±2.52)	<0.001
	D2	8.96 (±1.96)	11.53 (±2.75)	<0.001
	D3	8.07 (±2.33)	13.09 (±3.04)	<0.001
Mean platelet volume (fl) (Mean ± SD)	D1	11.41(±1.36)	7.87(±0.74)	<0.001
	D2	11.00(±1.20)	9.1(±2.29)	<0.001
	D3	10.22(±1.23)	11.69(±1.22)	<0.001
SOFA score		3.77 (±2.17)	7.87 (±2.65)	<0.001

## Discussion

This prospective study done among 100 patients diagnosed with sepsis found that the most common etiological factor of sepsis was respiratory infection (38%) followed by genitourinary (27%) and intraabdominal sepsis (21%). Previous studies had observed that the respiratory system was the predominant infective focus in 44% to 55% of patients [10-11]. Most of the patients in our study were from the 21-40-year age group (34%) followed by geriatric patients (>60 years) (30%). Patients with thrombocytopenia had a mean age of 49.06 years without any significant difference from the group with normal platelet count, which was in accordance with the findings of previous studies. In a previous study among ICU patients, 72% were male, with patients from the 21-40-year age group having predominant representation [12-13]. The male-female ratio was 1.08:1, which was supported by findings from previous studies [14-15].

in our study, 35% of patients had thrombocytopenia with a mean platelet count of 1.83±1.21 lakhs/mm^3^ on admission. However, in a study by Li et al., 48.7% of patients with sepsis experienced thrombocytopenia, which could be due to the different genetic compositions of the study population [16]. Another possible reason for the discrepancy may be that the reference study was conducted only among ICU patients, whereas we considered both ICU and IPD patients. Thrombocytopenic patients with sepsis exhibited a considerably more significant proportion of overall mortality, with a mortality rate of 30%. In addition, we observed a significant decline in mean platelet count toward the third day of admission among deceased patients, whereas survivors exhibited the opposite trend, which was similarly observed by a previous study [17]. Thrombocytopenic patients had a significantly longer mean hospital stay of 10.8±4.61 days, consistent with the results reported by Baughman RR et al. [18].

The mean SOFA score in patients with thrombocytopenia was significantly higher (7.4±3.1) than those with a normal count (3.7±1.92). A higher mean SOFA score among patients with thrombocytopenia with a statistically significant difference was also noted in a previous study [19]. Although the mean PaO2 was lower in patients with thrombocytopenia, this difference could not be established statistically. Similar observations were noted in a study done by Claushuis et al. [17]. Mean serum bilirubin was found to be slightly higher among patients with thrombocytopenia. This can be attributed to coagulopathy in sepsis, which was observed in multiple other studies that obtained similar conclusions [16-20].

We observed a statistically significant increasing trend in platelet distribution width (PDW) among deceased patients while survivors exhibited a trend of decline. As PDW demonstrates the size homogeneity of platelets, in deteriorating patients, elevated PDW can be attributed to anisocytosis due to increased immune platelet consumption. This finding was similar to what we observed in previous studies [21-22]. We also found that the mean platelet volume (MPV) increased till the third day of admission among patients who succumbed to death and decreased among survivors, similar to the findings of Pigozzi et al. [23]. Mean platelet volume (MPV) refers to the average size of platelets in the blood. Inflammatory markers, such as interleukin-6 (IL-6), IL-3, and thrombopoietin are associated with platelet production and size. In sepsis, the hyperactive immune system can destroy more platelets and suppress the bone marrow's ability to produce new ones. When platelet synthesis is reduced, the immature platelets grow larger and become more active, resulting in an increased MPV. This phenomenon is inversely related to platelet count. Therefore, patients with sepsis having higher MPV and PDW with lower platelet counts are likely to have a poor prognosis.

Limitations of the study

Our study has certain limitations that need to be acknowledged. First, it was a single-center; hospital-based study with a relatively small sample size, and it was conducted over a limited duration of one year. Therefore, to obtain a more comprehensive understanding of the association between platelet indices and sepsis, a larger study encompassing multiple centers, a larger patient cohort, and a longer follow-up period is required. Second, our study solely focused on mortality as an outcome measure and did not consider other critical outcomes such as major bleeding events, the need for mechanical ventilation, or renal replacement therapy. A more extensive investigation involving a broader range of outcomes would provide a more nuanced understanding of the relationship between platelet indices and sepsis.

## Conclusions

Our study demonstrated that patients with thrombocytopenia exhibit a higher SOFA score, which is significantly associated with patient outcomes. Additionally, platelet indices, such as platelet distribution width and mean platelet volume, serve as important prognostic markers among sepsis patients. These indices are simple and affordable, allowing for their serial assessment to aid in the prognosis of sepsis. Our findings reaffirm those of previous studies, which indicate that lower platelet levels are indicative of more severe disease and poorer patient prognosis. However, given the limitations of our study, future research involving a larger and more diverse patient population, longer follow-up periods, and more comprehensive outcome measures are needed to further elucidate this relationship.
